# The Effects of Menstrual Cycle Phase on Elite Athlete Performance: A Critical and Systematic Review

**DOI:** 10.3389/fphys.2021.654585

**Published:** 2021-05-19

**Authors:** Alice Meignié, Martine Duclos, Christopher Carling, Emmanuel Orhant, Peggy Provost, Jean-François Toussaint, Juliana Antero

**Affiliations:** ^1^Institute for Research in Medicine and Epidemiology of Sports (IRMES, EA7329), INSEP, Paris, France; ^2^Sport Medicine and Functional Explorations, University Hospital of Clermont-Ferrand (CHU), Clermont-Ferrand, France; ^3^Unité de Nutrition Humaine (UNH), Université Clermont Auvergne, INRA, Clermont-Ferrand, France; ^4^Fédération Française de Football, Paris, France; ^5^CIMS, AP-HP, Hôtel-Dieu, AP-HP, Paris, France; ^6^Université de Paris, Paris, France

**Keywords:** female, elite athlete, women, physiology, performance, individualization, injuries, menstrual cycle

## Abstract

**Background**: In elite athletes, training individualization is widely recommended to optimize competitive performance. Previous studies have evidenced the impact of hormonal fluctuations on different performance parameters among female athletes. While consideration of menstrual cycle (MC) phases as a parameter in training individualization strategies is necessary, systematic evidence identifying such impacts in elite athletes should be evaluated.

**Objective**: Systematically review publications that have investigated the link between MC phases and performance in elite female athletes. The overarching aim is to identify whether a consensus across studies exists enabling evidence-based recommendations for training individualization depending on menstrual cycle phases.

**Methods**: This review followed the Preferred Reporting Items for Systematic Reviews and Meta-Analyses (PRISMA) guidelines. Three major scientific publication databases were searched from inception until November 3, 2020. Studies included focused on the influence of physiological or psychological parameters throughout at least one phase of the menstrual cycle of elite athletes.

**Results**: A total of 780 search results were yielded and 26 references from a past bibliography were added manually. About 662 papers were reviewed of which 218 studies were assessed for eligibility. Of these, only seven (1%) precisely investigated the influence of a performance or physical parameter during at least one menstrual cycle phase. These seven studies included a total of 314 elite female participants (20.58 ± 1.91 years). Three used interviews, questionnaires or prospective analyses of reports. Four conducted several performance tests or included physical measures although only two performed tests during training or before/during competition. Among the seven studies, five performed hormonal testing through sampling of blood, saliva, or urine. The remaining relied on athletes’ menstruation diaries. The current evidence suggests a variable association between menstrual cycle and a few performance-related outcomes, such as endurance or power resistance, ligament stiffness, decision making skills, psychology, or competitiveness.

**Conclusion**: Different sports performance-related parameters are affected during the menstrual cycle among elite athletes, but the parameters themselves and the magnitude and the direction of the effects are inconclusive. Additional longitudinal and prospective studies to systematically monitor on-field performance parameters are urgently required in order to enable recommendations and training individualization in female elite athletes.

## Introduction

Female participation in Olympic Games has soared from the 1960 Games in Rome, where athletes only represented 11% of the total number of participants, to more than 45% at the 2016 Games in Rio. For the first time, there will be full gender parity in terms of athlete numbers at the Olympic Games Paris 2024. The number of women’s events also increased from 20% in 1960 to nearly 50% in Rio (International Olympic Committee, 2020), while their performances have substantially improved (Thibault et al., 2010). Research studies on sports performance determinants have also greatly increased, yet still mostly include male participants; females represent only 35% of the athletes studied (Costello et al., 2014). As such, the current body of research has generated greater knowledge on training strategies that have mainly aimed to enhance performance in male athletic populations, notably training individualization at elite levels, leading to more targeted training strategies and optimized performance (Kozina et al., 2015; Jiménez-Reyes et al., 2017). A one size fits all approach is impossible as athletes often respond differently to a given training stimulus, and the training load required for adaptation may differ significantly between sexes (Pedersen et al., 2019). Ideally, any individualized approach to preparation necessitates methods suited to each athlete’s needs in addition to technical and scientific expertise and resources.

**Figure 1:**
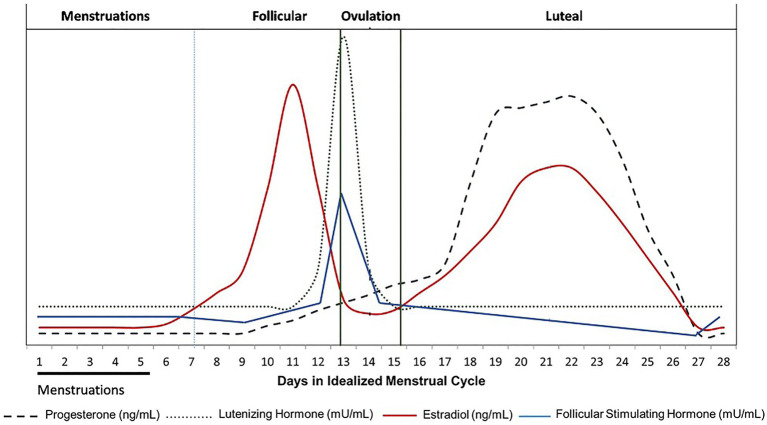
Hormone levels according to menstrual cycle (MC) phase. Changing concentrations of female sex hormones (progesterone, luteinizing hormone, follicular stimulating hormone, and estradiol) that characterize the four phases (menstruation, follicular, ovulation, and luteal) of the MC (adapted from Draper et al., 2018).

The frequent non-inclusion of female athletes in research studies has been justified by several potential cofounders including the menstrual cycle (MC) hormone variations. The fluctuations in hormones during the cycle generate a number of confounding variables impacting performance, thereby rendering difficulties in study design and subsequent interpretation of findings (Fox and Mathews, 1981; Lebrun, 1993). There is substantial interindividual variability leading to menstrual disturbance (amenorrhea, oligomenorrhea, irregular menstruation, anovulation…), and it seems to be more frequent among elite athletes (Redman and Loucks, 2005). Yet, the classical menstrual cycle length is 28 ± 2.4 days (Presser, 1974) and may be divided into four phases regulated by hormonal changes (Jurkowski et al., 1981; Figure 1). The cycle begins with the first day of menstruation when levels of estrogen and progesterone are low. This first phase is constituted by the follicular phase (FP), divided into two phases: menstruation (early follicular) and then the late FP. The late FP is the time between the first day of the period and ovulation, marked by the beginning of estrogen rise (Treloar et al., 1967). During the follicular phase, FSH and LH levels increase with FSH level higher than LH level in preovulatory phase (Simmen and Simmen, 2006). About 36 h after, the initiation of the LH peak (duration 48 h, mid LH cycle) ovulation occurs which constitutes the third phase of the cycle. Estrogen peaks just before ovulation and then drops shortly afterward. The luteal phase (LP; last phase) is the time between ovulation and the start of menstruation, when progesterone is produced (Vollman, 1977). During the follicular phase, FSH levels decrease, and LH levels are not fluctuating and are low. Ovulation constitutes the third phase of the cycle. Estrogen peaks just before ovulation and then drops shortly afterward. Progesterone levels also begin to increase. LH is released in a massive amount whereas FSH increases less. The last phase, the LP is the time between ovulation and the start of menstruation, when progesterone is produced at a higher level. It peaks and then drops at the end of this LP phase, concomitantly with an estrogen rise and fall. Progesterone promotes the secretary functions of the uterine endometrium, which prepares the endometrium for implantation of the fertilized ovum in the second half of the menstrual cycle. LH and FSH levels decrease. Within this continuous functioning, the gonadotropic axis (hypothalamus-pituitary) controls and modulates the final stages of follicular growth and ovulation under the influence of fluctuations in steroids and peptides secreted by the ovary (retrocontrol). Estradiol plays an important role in the development of primary and secondary female sexual characteristics. Subtle disruption of the balance between estradiol and progesterone during the menstrual cycle can affect multiple parameters, ranging from adverse symptoms including pain, fatigue, transient weight gain (water retention, increased food intake), sleep disturbance, and mood disorders (excitability, depressive tendency; Chabbert-Buffet, 2007). In addition to reproductive function, female sex hormones have significant nonreproductive physiological effects, including altering fluid regulation (Altemus et al., 2001) and modifying cardiovascular (Wagner et al., 2002), muscular (Nicklas et al., 1989), thermoregulatory (Hashimoto et al., 2014; Barnes and Charkoudian, 2021), and metabolic responses to different stimuli (Bailey et al., 2000). Constantini et al. (2005) established a list of components of sports performance that may be affected by the menstrual rhythm including cardiovascular, respiratory, brain function, response to ergogenic aids, orthopedics, and metabolic parameters, with subsequent implications for strength and aerobic and anaerobic performance. These parameters modulate training responses, adaptability, and performance (Oosthuyse and Bosch, 2010). The MC effects are highly dependent on athletes’ physiology, training level, and on the type of hormonal contraception when used. However, the effects of oral contraceptive pills tend to vary across studies and no general conclusions can be drawn (McNulty et al., 2020). Therefore, studies on contraception are not considered in this review.

While some female athletes feel a decrease in their physical capacity over the course of their menstrual cycle, Olympic medal-winning performances have nevertheless taken place during all phases of the menstrual cycle (Fox and Mathews, 1981; Fleck and Kraemer, 2004). Work in cross-country skiers showed that their best times were recorded in the postovulatory and postmenstrual phases, suggesting training loads should be selected according to cycle phase to optimize performance (Lebrun, 1993). Various studies have shown the influence of MC on performance in trained or untrained (Janse de Jonge, 2003) athletes but not in elite performers (McNulty et al., 2020). Elite athletes compete at international levels or in professional leagues; therefore, they are probably more sensitive to individualization of training.

In addition, in the few studies investigating exercise metabolism in women, participants were often tested in the early menstrual cycle phase (follicular phase) when hormones levels are at their lowest in order to minimize any potential impact (Fleck and Kraemer, 2004). This limitation has resulted in a misunderstanding of the extent to which hormones influence the specificities of female physiology, from cardiovascular to autonomic nervous systems, thermic stress responses (Hashimoto et al., 2014; Barnes and Charkoudian, 2021) or energetic metabolic pathways (Tarnopolsky, 2008; Fu et al., 2009), and even cell-mediated immunity (Suzuki and Hayashida, 2021). For instance, estrogen can enhance endothelium-dependent vasodilatation (Chan et al., 2001) or when estrogen level are low it increase cardiovascular responses to stress (Altemus et al., 2001). Progesterone have a central thermogenic effect, modulated at the level of the preoptic/anterior hypothalamus (Charkoudian and Johnson, 2000). Hence, MC can be seen as a potential performance determinant yet remains neglected as hormonal fluctuations are not appropriately considered in the individualization of women’s training. Nevertheless, new studies have emerged recently including female athletes and investigating training adaptations according to their MC (Knowles et al., 2019; Blagrove et al., 2020). Planning recommendations have been forwarded and media attention has subsequently increased around this subject.

Accordingly, this study aimed to systematically review research protocols that have investigated the link between MC phases and performance in elite female athletes in an attempt to provide evidence-based recommendations for training individualization related to performance. Studies directly investigating the effects of MC phases on elite performance parameters (such as endurance or power resistance, decision making skills, symptoms linked to MC, ligament stiffness, and/or competitiveness) were selected and discussed.

## Materials and Methods

### Protocol and Registration

The present review was conducted according to the Preferred Reporting Items for Systematic Reviews and Meta-Analyses (PRISMA) guidelines (Liberati et al., 2009). The study was preregistered under the international prospective register of systematic reviews and is being assessed by the editorial team (PROSPERO; ID: 226662).

### Search Strategy

This review focuses on research studies in elite athletes directly investigating the effects of MC phases on performance parameters such as endurance or power resistance, symptoms linked to MC, ligament stiffness, decision making skills, and/or competitiveness (desire to compete and training motivation). To identify eligible papers, a primary search was conducted in the PubMed, Sportdiscus, and Researchgate databases, using a well-defined search strategy that was formulated *a priori*. The search was conducted using a Boolean strategy following search terms: (“menstrual cycle” OR “menstrual phase” OR “luteal phase” OR “follicular phase”) AND (“medal” OR “performance”) AND (“elite” OR “athlete” OR “professional” OR “olympic” OR “high-performance”). No restriction was placed upon date of publication. To identify any articles that may have been missed during this literature search, the reference list of eligible papers was carefully checked. No limit on year of publication was set, and the final search was updated to November 3, 2020. The complete search equations for all databases are available in the Supplemental Material. In addition, the search was complemented with references harvested from a bibliography of included studies and overview articles.

### Study Selection

Studies that reported at least one performance parameter (either athletic or mental) related to the menstrual cycle or at least one MC phase (e.g., luteal, follicular or menstrual phase) and elite athletes were included. We defined elite as an athlete that competed at national or international level and excluded studies qualifying women as only “trained,” “untrained,” or “recreational” (Swann et al., 2015). No age restrictions were used for this review. Excluded studies also concerned those not distinguishing between the different MC phases nor evaluating a physiological parameter. Unpublished studies, non-peer reviewed publications, review articles, abstracts, and publications not available in English were excluded.

### Eligibility Criteria

**Figure 2:**
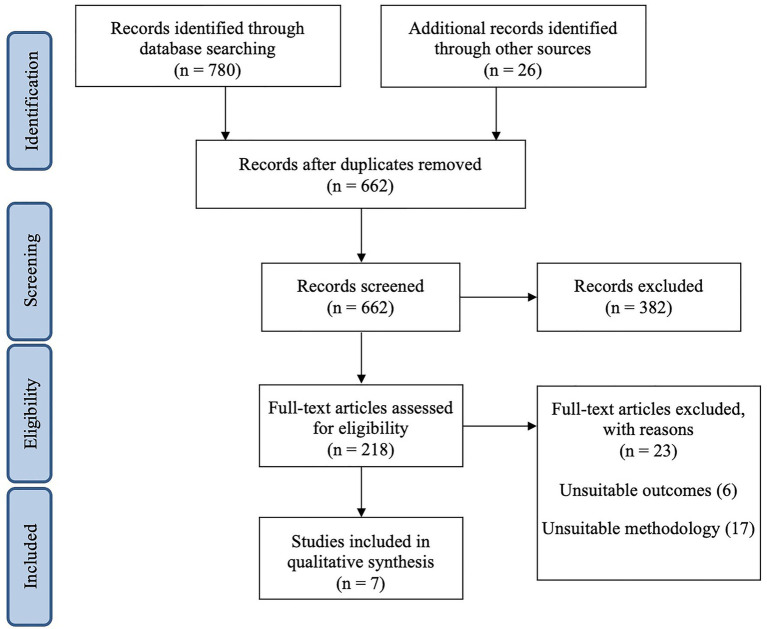
Flow diagram of bibliographic process based on Preferred Reporting Items for Systematic Reviews and Meta-Analyses (PRISMA) guidelines (Liberati et al., 2009).

The search strategy is detailed in Figure 2. All manuscripts initially considered relevant by title and abstract were eligible for inclusion. The full text of the manuscripts was obtained to ascertain whether they satisfied the following inclusion criteria, detailed according to the PICO standard:

P (population): athletes (high-level, national, or international and practicing any sports discipline);
I (intervention/exposure): having performed any form of study (questionnaires, laboratory or on-field physical tests) including at least one MC phase;
C (comparison): studies including athletes comparing performances outcomes at different MC phases;
O (outcome): link between MC phases and performance in elite athletes.

Other inclusion criteria concerned the protocol design (questionnaire, physical tests in laboratory or on-field, etc.), language (all languages available), and time filter (none applied).

In order to broaden the research, the reference section of each selected articles was searched manually to identify other relevant articles. Target journals were also hand-searched. Furthermore, a gray literature search using Google Scholar and a search of dissertations databases were performed.

### Data Extraction

The following parameters were extracted from the collected studies by the authors (AM, JA, MD): (a) author, (b) country and year of publication, (c) sport, (d) number of athletes included, (e) participant age, (f) type of study (e.g., questionnaire, physical tests in laboratory conditions or on-field), (g) method of MC phase evaluation, (h) outcomes measures, (i) test performed, (j) results, and (k) statistical significance. Data were extracted from the included studies and collated into a table for further quantitative and qualitative analysis (Supplementary Material).

### Critical Appraisal of Methodological Quality in Individual Studies

Two reviewers independently appraised the methodological quality of included studies. Given that the eligibility criteria included both interventional and observational study designs, the Downs and Black protocol was used due to the robustness of the checklist that includes quality of reporting, internal validity (bias and confounding), external validity, and statistical power (Downs and Black, 1998). This checklist relies on 27 questions to assess the methodological quality of studies and has been used in reviews involving populations. For some studies, several questions of the Downs and Black protocol (for instance 4, 8, 13, 14, 15, 19, 23, and 24) were not deemed relevant as these were dedicated to experimental studies rather than an observational research design. A binary score was used for all items [0 = no/unable to determine (UTD); 1 = yes], except for item 5, which used a larger scale consistent with the original Downs and Black checklist (0 = no/UTD; 1 = partial; 2 = yes). However, scoring for question 27, relating to statistical power, was modified to award one point for a “yes” answer, indicating the authors had reported a sample size or power analysis, or zero points for a “no” answer, indicating they had not. This revised scoring approach for question 27 replaced the original six-point scoring scale, ranging from zero to five points, and reduced the maximum possible raw score for the Downs and Black checklist to 28, from the original 32 points.

The use of this modification to the Downs and Black checklist was not considered to have impacted the analysis of study quality: it meant that the included studies were not assessed against criteria for which they had never been designed. This method has previously been used (Alla et al., 2009; Freckleton and Pizzari, 2013; Farley et al., 2020).

Owing to the modifications made to the Downs and Black checklist, discussed above, all scores were first converted to percentages to enable grading using the approach of Kennelly (2011). On this basis, the grading criteria applied when rating the methodological quality of the included studies were as follows: Downs and Black total score <45.4%, “poor” methodological quality; 45.4–61.0%, “fair” methodological quality; and >61.0%, “good” methodological quality. Disagreement was resolved by discussion or by consulting a third author (MD).

## Results

### Study Selection

The initial search from the three databases returned 780 results; 26 references from a past bibliography were added manually (Supplementary Material). According to the aforementioned exclusion criteria, 662 papers were reviewed (Figure 2) including 218 publications relating to sports and MC phases. Among these, 30 studies on elite athletes were conserved for further investigation. Around 23 papers either focused on MC as a whole, not distinguishing each phase, or examined a non-performance parameter (e.g., study of hormonal contraception). In total, seven publications investigating different performance parameters in female elite athletes throughout the different phases of the MC were taken forward for full analysis.

Collectively, the seven selected studies included 314 female elite athletes aged 20.58 ± 1.91 years. According to classification of Sundgot-Borgen and Larsen (1993), sports included are endurance sport (triathlon, swimming), weight-class sports (judo, taekwondo), and ball games (soccer, rugby, netball, handball, and volley-ball). On average, the publications included 28 athletes but only nine who participated in studies involving physical testing and not gathering of information using questionnaires. Study durations varied from 8 weeks to 7 months with an average of 3 months. The only prospective study (Myklebust et al., 1998) used data collected from 1993 to 1996.

### Quality Assessment and Risk of Bias

The results of the critical appraisal of the methodological quality of the seven included studies, using the modified Downs and Black checklist (Downs and Black, 1998), are shown in Supplementary Table 1 as raw scores. Questions from the checklist that were ignored as planned due to a lack of relevance to the research designs of the included studies are stated as “not relevant.” One study was graded as being of “fair” methodological quality (Tounsi et al., 2018), while the remaining six were graded as being of “good” methodological quality (Myklebust et al., 1998; Kishali et al., 2006; Julian et al., 2017, 2020; Crewther and Cook, 2018; Statham, 2020). The mean (±SD) percentage score for methodological quality of the included studies was 69.0% (±7.1%), with a range of 60.7 (Tounsi et al., 2018) to 83.3% (Myklebust et al., 1998).

Noteworthy limitations identified among the studies were: participants that were not representative of the entire population from which they were recruited, no adequate adjustment for confounding variables, such as training load, or recovery status was performed, and only one study demonstrated adequate power analysis for its study sample.

### Outcome Variables

Among the seven papers selected, four studies analyzed athletes’ physical performances (Julian et al., 2017, 2020; Tounsi et al., 2018; Statham, 2020). All testing protocols were performed in at least one of the MC phases, that is, either in the LP (*n* = 6, Myklebust et al., 1998; Kishali et al., 2006; Julian et al., 2017, 2020; Crewther and Cook, 2018; Tounsi et al., 2018) and/or FP (*n* = 7, Myklebust et al., 1998; Kishali et al., 2006; Julian et al., 2017, 2020; Crewther and Cook, 2018; Tounsi et al., 2018; Statham, 2020). Kishali et al. (2006) also focused on the difference between morning and afternoon in the LP or FP. One field-based study analyzed external load data collected directly in match-play conditions using Global Positioning System devices (GPS) in a group of elite soccer players (Julian et al., 2020). Information included the distances covered per minute at varying intensities. Two other studies in soccer players included data from field tests of physical performance such as 3 × 30 m sprints to evaluate sprinting capacities, a counter movement jump or a Five-Jump Test to assess lower limb power or the Yo-Yo Intermittent endurance test to measure endurance capacities (Julian et al., 2017; Tounsi et al., 2018). The remaining study employed the Cambridge Gambling Task (CGT) to test decision making (Statham, 2020).

The three other studies relied on questionnaires given to athletes (Kishali et al., 2006; Crewther and Cook, 2018) or prospective analyses in an epidemiological study of anterior cruciate ligament (ACL) injuries (Myklebust et al., 1998). Kishali et al. (2006) asked 21 questions about anthropometric and descriptive data about menstrual cycle including status of participation to training and competition, status of place in competition during menstruation cycle or regularity of the menstruation, presence of dysmenorrhea, and the use of drugs such as pain relieve during menstruation. Crewther and Cook (2018) developed a short competitiveness questionnaire with two summary questions: desire to compete (from 1 = I have no desire to compete up to 7 = I feel extremely competitive) and training motivation (from 1 = I have no motivation to train up to 7 = I am extremely motivated to train).

Regarding the MC phase, two performed serum analysis (estrogen and progesterone, Julian et al., 2017, or only progesterone, Tounsi et al., 2018), one used urine ovulation kit (Statham, 2020) and one used both serum and urine ovulation kit (Julian et al., 2020). All studies evaluated hormones levels during at least three MC. Two studies did not quantify hormones levels. One based their protocol on a declarative menstrual diary (Kishali et al., 2006) and the other was a prospective study based on athletes declarative menstrual diary (Myklebust et al., 1998). One performed salivary testosterone measurements (Crewther and Cook, 2018) to validate the declarative menstrual diary of athlete.

A total of six (86%) of the seven articles identified a variation in performance between the LP and FP and only one focused on the impact of different parameters at ovulation (Crewther and Cook, 2018; Supplementary Table 2). Julian et al. (2017, 2020), revealed a significant reduction in maximal endurance performance during the LP phase in elite soccer players based on a protocol lasting 8 weeks. In contrast, no significant reduction in jumping or sprint performance during MC was observed. Likewise, match physical performance metrics (total distance covered in each intensity zone, number of high intensity and sprinting bouts) in female soccer players were not significantly influenced by MC (Julian et al., 2020). Tounsi et al. (2018) revealed that repeated-sprinting and jumping performance were better in the afternoon rather than in the morning irrespective of the MC phase, while soccer-specific endurance did not show such a morning-to-afternoon difference during each MC phase. Injury rates were also investigated and notably ligament stiffness. ACL ruptures were more frequent during the weeks prior to or after the onset of menses in handball players (Myklebust et al., 1998).

Statham (2020) demonstrated changes in cognitive behavior through evaluation of the following parameters: impulsivity, risk taking, response time, and error rates by doing different CGT. The authors suggested that impulsivity was significantly influenced by MC phase, being greater during the menstruation phase compared to other phases. The authors also conducted interviews and observed that athletes and their coaches understood little about the MC and thus, had preconceptions that negatively impacted performance during the MC phase. Also, Crewther and Cook (2018) demonstrated that competitive desire and training motivation both peaked around ovulation and salivary testosterone (sal-T) concentration and its relationship with competitiveness was stronger among high-performing athletes (Crewther and Cook, 2018). Kishali et al. (2006) investigated psychological metrics around the MC. Between menstruation periods, the athletes stated *via* questionnaires that they felt better in the first 14 days compared to the second half of their MC. Most of the athletes said that they had a painful menstruation period, and that their pain decreased, while in competition (Kishali et al., 2006) and their physical performance was not affected by their menstrual period.

## Discussion

A recent review has shown that responses to physical training can be improved in female athletes, when adjusted to MC phases (McNulty et al., 2020). However, insufficient evidence has arguably been provided so far to warrant systematic implementation of training programs specific to elite female athletes. Yet, performance oscillations during MC phases are not yet fully understood because studies have investigated the influence of the MC as a whole on elite performance and not the different phase of the MC. These assess different performance parameters at any time of the menstrual cycle, but do not evaluate fluctuations of the parameter by selecting different phases of the MC. This article systematically reviewed research protocols investigating the link between MC phases and performance in elite athletes.

Two main findings were identified: First, there is generally a lack of knowledge regarding the relation between MC phases and performance in elite athletes. Only seven studies have investigated this link of which four relied on objective measurements of physical and performance outcomes. In addition, several methodological issues were identified. Second, performance outcomes appeared to remain stable along the MC and it is not clear whether there is an optimal phase for performance. Some physical or cognitive capabilities were better during the LP than the FP (e.g., endurance performance) or during ovulation (e.g., impulsivity, competitiveness). Others did not seem to be influenced by the MC (e.g., lower limb power, sprinting, risk taking, and response time).

The present review shows that in general too few studies on elite female athletes currently exist. Since elite athletes endure intensive training and competition schedules, the implementation of research programs is difficult. Therefore, most studies are performed on non-elite athletes albeit providing some clues for elite athlete populations (Oosthuyse and Bosch, 2010; Bruinvels et al., 2017). However, conflicting evidence exists from studies in elite vs. non-elite athletes. Conversely to findings reported by Julian et al. or Tounsi et al. on sprinting capabilities in elite athletes, past studies found that the intensity corresponding to the lactate threshold was higher during the LP than FP reporting lower blood lactate concentrations during exercise in untrained or non-elite trained athletes (Sung et al., 2014; Lane et al., 2015). Also, time to exhaustion at 90% of maximum power output was doubled in the LP phase compared with the FP phase (Jurkowski et al., 1981), while contrasting results were reported in other studies (Bailey et al., 2000). Investigations reporting better performance, in the LP reported a higher estrogen-progesterone ratio (Oosthuyse and Bosch, 2010). Another study regarding sprint performance in non-elite athletes reported that hormonal fluctuations due to the MC did not interfere with maximal intensity whole body sprinting and the metabolic responses to such exercise (30-s sprint on a non-motorized treadmill interspersed with a 2-min rest in three phases of the MC; Tsampoukos et al., 2010).

Regarding physical outcomes, in line with the prospective study on ACL injuries of Myklebust et al. (1998), some studies investigating elite and non-elite but trained athletes showed that hormone levels in females are related to increased knee joint laxity and decreased stiffness at ovulation (Belanger et al., 2013; Herzberg et al., 2017). Research has demonstrated a significant negative correlation between estradiol (assessed *via* radioimmunoassay) and ACL stiffness (assessed with knee arthrometer) and a significant positive correlation between estrone and ACL stiffness near ovulation (Romani et al., 2003). Park et al. (2009) observed a reduction in knee stiffness of approximately 17% during ovulation using a standard knee arthrometer. Ligament stiffness, although also a risk factor for injuries, was included in this review considering its potential association with performance outcomes, since higher levels of lower-body stiffness are related to better physical performance (Pruyn et al., 2014) and lower risk of counter-performances. Studies suggest that the presence or absence of Premenstrual symptoms (PMS) or menstrual syndrome symptoms may have an effect, possibly through an action on the stretch-shortening cycle of tendons and ligaments (Giacomoni et al., 2000).

Indeed, PMS such as fluid retention, weight gain, mood changes, and dysmenorrhea were associated with performance decreases and physical capacities in female soccer players (Lebrun, 1993; Foster et al., 2019). The availability of evidence suggests that post-match sleep deprivation may result in depreciated cognitive functions in the following morning, which may affect attention and decision-making skills during ensuing training sessions, potentially resulting in an increased injury risk (Nédélec et al., 2015). Knowing that hormonal fluctuations have an impact on sleep quality (Nowakowski et al., 2013), a deleterious effect on decision-making skills might occur as reported in Statham (2020).

Other studies have focused on exercise-induced growth hormone (GH, an anabolic hormone that increases with acute exercise) responses during MC, proportionally to exercise intensity (Pritzlaff et al., 1999). The secretory response of GH to a resistance exercise session in women is greater during LP than during FP (Bird, 2013). Following a prolonged bout of aerobic exercise, plasma levels of biologically active free testosterone (androgenic responses) are elevated during LP, but without any observable change during FP (Lane et al., 2015). This suggests a potential stronger anabolic reaction in response to exercise during LP, which could be pertinent during strength training or for recovery following heavy load training (Sung et al., 2014).

To date, studies are scarce and only a few firm conclusions can be established regarding which, when, and how performance components may be affected by hormonal variations. The impact of such variations is unknown since this may be concealed by other strong performance determinants such as training load, injuries, or recovery strategies, including nutrition or kinesiotherapy. The complexity of performance renders achieving consensus on the influence of performance parameters during MC even more difficult. Each study reviewed here was designed specifically to the population studied and drew a specific conclusion for a given and controlled environment. While this is interesting for the potential individualization of training for the population studied, recommendations cannot be provided to athletes in general. Another limitation is that performances are often evaluated “artificially.” Studies testing athletes in a laboratory setting (e.g., wattbikes, treadmill) or physical performance evaluated through physical tests (Five-Jump Test to assess lower limb power or the Yo-Yo Intermittent endurance test for instance) that may not realistically represent situations on the field. Findings under controlled settings are neither fully transposable nor beneficial to elite sport and one cannot, based on current knowledge, prescribe a training strategy that considers athletes’ menstrual cycle.

Consequently, links between exercise performance and MC could not be consistently identified from the present findings. Yet, most researchers have based their protocols on declarative menstrual diary to assess the MC phase of each athlete. There is no denying that performing blood sampling is invasive for an athlete, even recreational one, but strong conclusions cannot be drawn if hormones and performances are not precisely quantified. Absolute measures of key menstrual hormones are essential in research to create accurate and individualized athlete hormonal profiles that can be correlated to markers of performance (e.g., strength and plyometric capabilities), injury, and training response (Julian and Sargent, 2020). New less invasive solutions should be developed and used to analyze hormonal fluctuations, such as salivary, blood-drop or even dry blood-spot testing (Janse DE Jonge et al., 2019). Also, studies are generally too short to comprehensively evaluate hormonal profile of athletes. Athletes should be followed during at least 3 months over three full MC to avoid variability of menstrual cycle and possible troubleshooting (Fehring et al., 2006).

Therefore, evidence-based recommendations for training individualization according to each MC phase to improve performance in female elite athletes are not currently available. Especially since a variation of concentrations of ovarian hormones between subjects and from day to day within subjects can exist during any particular menstrual phase. Indeed, effects of ovarian hormones on metabolism or physiology have been reported from many laboratory studies and suggest repercussions for exercise performance in eumenorrheic women (Oosthuyse and Bosch, 2010). However, the influences of the ovarian hormones on the various metabolic pathways are complex and often tissue specific and athlete specific.

Based on the difficulties highlighted in this review, we suggest a need for additional longitudinal and prospective studies. Robust statistical methods may establish and validate causal links, quantify impacts, and make reliable recommendations that can guide evidence-based future training individualizations. Studies should involve a sufficient number of athletes in order to obtain conclusive results. Indeed, each female is different from another and does not respond similarly to training throughout the menstrual cycle. Studies have already shown the existence of divergent hormonal profile (Lanhers et al., 2020). This review reinforces the fact that there is a variety of athlete responses to physical tests. All the publications reviewed highlighted individual responses to menstrual issues. These findings emphasize the need for researchers and support staff to undertake menstrual cycle profiling and monitoring and continue to develop awareness, openness, knowledge, and understanding of MC.

## Conclusion

In addition to being limited in number, studies examining the link between menstrual cycle and performance at an elite level are typically based on cross-sectional test designs generally conducted in laboratory settings or assessed subjectively *via* questionnaires. The few studies available are not easily transposable to the elite field. Thus, we cannot formulate solid conclusions regarding the impact of the MC for elite athletes.

The impact of not knowing the MC effect on performance is 2-fold: (1) elite female athletes will continue to suffer the effect of MC instead of this being used as a potential advantage and (2) studies are still done on men athletes because we want to get rid of the unknown impact of MC on performance outcomes. Thus, the significant gap in understanding the extent to which MC impacts high-performance is perpetual and can explain why it is still perceived as a barrier to training and performance as well as influencing injury risk and well-being.

To sum up, there is a clear lack of evidence-based recommendations on training individualization according to the menstrual cycle. In our opinion, a better understanding and mastery of the relation between the menstrual cycle and athletes’ training responses could subsequently provide a sizeable performance advantage.

## Data Availability Statement

The original contributions presented in the study are included in the article/Supplementary Material; further inquiries can be directed to the corresponding authors.

## Author Contributions

AM performed the search, quality assessment, and data analysis. JA contributed to the search of databases. AM and JA carried out the drafting of the manuscript. All authors contributed to the manuscript revisions and approved the submitted version.

### Conflict of Interest

The authors declare that the research was conducted in the absence of any commercial or financial relationships that could be construed as a potential conflict of interest.

## Supplementary Material

Supplementary Table 1 Critical appraisal score and levels of evidence of each included study using modified Downs and Black critical appraisal checklist (Downs and Black, 1998). UTD, unable to determine; N/A, not applicable.

Supplementary Table 2 Synthesis of analyzed publications that meet all inclusion criteria. Results expressed as means ± SDs. Kg, kilograms; m, meters; min, minutes; s, seconds; #: number.
